# Clinical and Socio-Demographic Characteristics of College Students Exposed to Traumatic Experiences: A Census of Seven College Institutions in Northeastern Brazil

**DOI:** 10.1371/journal.pone.0078677

**Published:** 2013-11-13

**Authors:** Liana R. Netto, Patrícia Cavalcanti-Ribeiro, Juliana L. Pereira, José F. Nogueira, Lene L. Santos, Sidnei B. Lira, Gisela M. Guedes, Carlos A. Teles, The Trauma, Anxiety Disorders Study Group (TADSG) -. UFBA, Karestan C. Koenen, Lucas C. Quarantini

**Affiliations:** 1 Programa de Pós-graduação em Medicina e Saúde, Faculdade de Medicina da Bahia, Universidade Federal da Bahia, Salvador, Bahia, Brazil; 2 Departamento de Neurociências e Saúde Mental, Faculdade de Medicina da Bahia, Universidade Federal da Bahia, Salvador, Bahia, Brazil; 3 Universidade Estadual de Feira de Santana, Feira de Santana, Bahia, Brazil; 4 Department of Epidemiology, Mailman School of Public Health Columbia University, New York, New York, United States of America; Federal University of Rio de Janeiro, Brazil

## Abstract

**Background:**

Epidemiological studies show that most of the adult population will be exposed to at least one potentially traumatic event in the course of his/her life; adolescence and early adulthood are the most vulnerable periods of life for exposure to traumatic experiences (70% of their deaths are due to external causes). Posttraumatic Stress Disorder is characterized by the development of dysfunctional symptoms that cause distress or social, academic, or occupational impairment, as result of exposure to a traumatic event. The aim of this multicentric study is to establish the proportion of college students, within seven institutions in Northeastern Brazil, who were exposed to traumatic experience and met PTSD criteria.

**Methods/Design:**

A one-phase census protocol of seven college institutions in three metropolitan regions in Northeastern Brazil was performed (April to July 2011). All students aged 18 years or older, matriculated and attending their first or final semester were eligible. The self-applied protocol consisted of a socio-demographic questionnaire and the following scales adjusted to Brazilian Portuguese standards Trauma History Questionnaire (THQ), PTSD Checklist-Civilian (PCL-C), Impulsivity Scale (BIS-11). Data were entered into SPSS 17.0.

**Results:**

2213 (85.5%) students consented to participate, and completely filled in the protocols. Of these, 66.1% were woman, mean age 23.9 (SD 6.3), 82.7% were single, and 57.3% attended university outside their native cities. The total PTSD prevalence was 14%, and the median for frequency of trauma exposure was 5 events.

**Conclusion:**

A high frequency of exposure to violence, as well as a high rate of PTSD, suicide attempts, and high-risk sexual behavior was found in Brazilian college students. This highlights the importance of effective public health actions in relation to the prevention and treatment of PTSD and other dysfunctional behaviors resulting from traumatic exposure in young individuals, usually an at risk population for violence and traumatic situations.

## Introduction

Epidemiological studies show that most members of the adult population will be subject to at least one potentially traumatic event in the course of their lives [1], [2]. Among all stages of life, adolescence and early adulthood are the greatest periods of vulnerability for exposure to traumatic experiences, whereas 70% of adolescent deaths are due to external causes (68.43% between the ages of 10 and 19 years), and likewise for young adults (70.41% between 20 and 29 years) [3].

Greater impulsiveness and assuming of high-risk behavior, due to an imbalance in neurofunctional regulation between late prefrontal cortex development (responsible for executive function and inhibitory control) and increased responsiveness in the *nucleus accumbens* (responsible for novelty seeking and immediate gratification), occurs in the beginning of adult life [4], and may contribute to members of this age group exposing themselves to interpersonal violence.

The vulnerability of college students to exposure to traumatic experiences may also be seen as a result of the abrupt transition to independent living. This is often associated with isolation and the loss of support from home [5], which has been demonstrated by previous research regarding involvement in violent situations [6], and can be even more dramatic where the setting favors exposure to violence: rates of violence are particularly high in the Americas, where the average homicide rates for the years 2000–2004, estimated at 17.8 homicides per 100,000 inhabitants, were the highest in the world [7]. In many international comparisons, performed using data from the World Health Organization, Brazil has always occupied one of the top positions among Latin American countries in terms of high homicide rates [2].

In 2000, violent deaths were predominantly caused by homicides [8], [9], with the risk 12 times higher for males (53/100,000) than for females (4/100,000) [10]. Ninety percent of these deaths were perpetrated with firearms in urban areas [11], and since 1998 (when the first official survey was made to map violence in Brazil) to the present day, the youth population retains a high prevalence among homicide cases representing a major unresolved problem for the country’s policies.

In spite of the history of violence in Brazil, there are no previous local populational data about the prevalence of PTSD, including among college students. The aim of this study is to describe clinical and socio-demographic characteristics of college students at seven college institutions in Northeastern Brazil who have been exposed to traumatic experiences.

## Methods/Design

### Ethical Aspects

This study has been approved by the Ethics Review Board of Bahia (CEP/COM/UFBA- process number 227/2010) and Paraíba (CEP/Fac. Sta. Maria, 17-02-2011). Participants were informed about research procedures and risks. They signed an informed consent form and received a copy of it. The informed consent form included a list of addresses and phone numbers of public institutions and services supporting mental health – psychiatric and psychological – that could handle any demands which the questionnaire may provoke. All questions were answered and any points of uncertainty were clarified. If the potential subject did not provide informed consent, he/she was excluded from the study.

### Study Design

This study constitutes a one-phase research project that aims to explore key aspects of traumatic experiences in college students from seven institutions in Northeastern Brazil.

### Setting

Northeastern Brazil contains 27.8% of the Brazilian population, totaling 53,081,950 inhabitants and became, in 2010, the region with the second-highest concentration of undergraduate students in the country (19.3% of all Brazilian students) [12]. Of these, 31.8% are matriculated at federal universities, 31.4%, at state universities and 36.8% at private institutions [12].

The majority of the population is concentrated in urban areas (73.1%), and 80.9% are 29 years or younger [13]. Data from the Brazilian Institute of Geography and Statistics (IBGE) showed that in 2010 the Brazilian average annual income was U$ 3.808,28, while the average annual income in Southeastern Brazil was U$ 4.683,72 and the average annual income in Northeastern Brazil was U$ 2.277,12 [13].

### Sampling Procedure

Seven college institutions were selected for reasons of convenience in three urban areas of Bahia and Paraiba. In order to select representative college institutions, we sought to include three public (2 federal and 1 state) and four private colleges in Northeastern Brazil; thereby capturing a broad geographic profile of Northeastern college students, considering that students, very often, attend university outside their native cities [14].

The study included all students matriculated at the university and attending their first or final semesters in all coursework from the 7 college institutions, aged 18 years or older, and who consented to participate in the study by signing the consent form.

### Measurements

The self-applied protocol included a fully structured socio-demographic questionnaire along with three scales which have been widely applied in epidemiological surveys. All of the scales had been previously translated and adapted to Brazilian Portuguese. All participants answered the full assessment anonymously, which lasted approximately 20–30 minutes.

#### Socio-demographics

The socio-demographic questionnaire included gender, age, marital status, employment status, parents’ educational level, annual family income, migration history, and parents’ marital status.

#### Mental health

psychiatric symptoms assessed in the study are:

- PTSD (assessed through the PTSD Checklist- PCL-C [15]): the instrument is comprised of 17 items based on the diagnostic criteria of the DSM-IV for PTSD. Thus, the first 5 items refer to the re-experience symptoms group (criterion B), the next 7 items refer to the emotional avoidance/numbing group (criterion C), and the last 5 items address the hyperarousal group (criterion D). In the instructions on how to fill-in the PCL-C, the subject was instructed to anchor their answers to the worst trauma he/she had experienced according to the Trauma History Questionnaire, and was asked to report how much he/she has been troubled by the listed problems and complaints in the past month (not at all, a little bit, moderately, quite a bit, or extremely). The Brazilian version of the PTSD Scale (PCL-C) has received a transcultural adaptation [15]–[17] which has become widely accepted. The diagnosis was made by combining two methods, in order to improve accuracy and ensure that an individual has the necessary pattern of symptoms with sufficient severity as required by the DSM-IV: the first method requires that the individual matches at least one B item (questions 1–5), at least three C items (questions 6–12) and at least two D items from the DSM-IV (questions 13–17). Symptoms rated as “moderately” severe or greater are considered clinically meaningful [18]. The second method determines whether the total severity score equals or exceeds a given cut-off point. A total symptom severity score (range = 17–85) can be obtained by taking the sum of the scores from each of the 17 items. Based on Adkins et al. (2008) [19], which used a similar setting (civilian trauma-exposed undergraduates) to explore and compare the psychometric properties of seven self-reported measures of PTSD, the adopted cut-off point for PCL-C was ≥45. According to the authors, this was the optimally efficient cut-off score previously found for this population, which yields a sensitivity of .78 and specificity of .92, positive predictive value of .54, negative predictive value of .97, efficiency of .91, quality of sensitivity of .49, quality of efficiency .59, and quality of specificity .74.
- Impulsivity (assessed through BIS 11 [20]): this 30-item self-administered scale assesses the presence of impulsive behaviors from the theoretical model proposed by Ernst Barratt through 3 factors: motor, attentional and lack of planning [21], it being the most widely used scale for clinical and research propose. The three factors are randomly distributed throughout the scale, and the answers are given according to 4 options: 1. rarely/never, 2. sometimes, 3. often, 4. almost always/always
- Alcohol, tobacco and illicit psychoactive substance use, assessed through five questions which aim to investigate the substance use pattern: first use, current use, frequency, quantity, interpersonal consequences (fights, accidents or other high-risk situations that occurred during substance use).
- Sexual risk behaviors (assessed through a single Yes/No question about condom use during any sexual relationship with a non-stable partner).
- Suicide (assessed through the question of how many times one has attempted to commit suicide).

#### Exposure to traumatic events

Assessed through the Trauma History Questionnaire (THQ) [22], which is a list of 24-items, including 23 events that could be considered potentially traumatic and 1 item that allows subjects to report on any personal experiences that were not captured in the other 23 items. Information on the frequency and age(s) at the time(s) of exposure was also obtained. At the end, participants are asked to select, from the items identified on the THQ, the event they found the most distressing. The Brazilian version of THQ has received a transcultural adaptation [23] which is widely accepted.

### Procedures

The preparative procedures for the collection began in October 2010. Data was collected from April 2011 until July 2011. For a more complete explanation of the investigation flow, see Figure S1.

Efforts were made to reach all students in their first or final semester, matriculated at the university and attending coursework from the 7 college institutions, such as: revisiting classrooms, awaiting students’ arrival, and rescheduling visits.

Data regarding the profile of absent students (25.4% of students matriculated at the university and not encountered in classes) were acquired in order to compare with those active students enrolled in the study.

The training course for researchers consisted of a 10-hour theoretical module, followed by a field application conducted by the authors. Regular meetings with supervisors were carried out in order to give clarifications and standardize the interview procedures.

### Data Analyses

1. Description of participants’ characteristics by means of univariate analyses: age, gender, marital status, academic performance, parental educational level, origin (being local or from another city), attending semester and family income.
2. The prevalence of estimated PTSD, alcohol misuse and other illicit psychoactive substance use, suicide attempts, high-risk sexual behavior and exposure to other traumatic life experiences were made using crosstabulation.

## Results

The analyses were conducted among a population of 2213 subjects (56.9% matriculated at the university in their first semester, and 43.1% in their final one), of whom 57.3% were attending university outside their native cities. The students were mostly woman (66.1%), mean age 23.9 (SD 6.3), and single (82.7%). (Table 1) The total PTSD prevalence was 14%, among which the most impulsive were the most affected (56.1% of PTSD subjects).

**Table 1:** Socio-demographic characteristics of college students exposed to traumatic experiences in Northeastern Brazil.

Socio-Demographic Variables		Total N (%)	Without PTSD N (%)	With PTSD N (%)
**Gender**	**Female**	1412 (66.1)	1183 (83.8)	229 (16.2)
	**Male**	725 (33.9)	653 (90.1)	72 (9.9)
**Age**	**≤22 years**	1138 (51.4)	993 (87.3)	145 (12.7)
	**>22 years**	1075 (48.6)	910 (84.7)	165 (15.3)
**Marital status**	**Single**	1789 (82.7)	1548 (86.5)	241 (13.5)
	**Married**	342 (15.8)	290 (84.8)	52 (15.2)
	**Divorced**	27 (1.2)	18 (66.7)	9 (33.3)
**Origin**	**Local**	844 (42.7)	732 (86.7)	112 (13.3)
	**Non-local**	1131 (57.3)	970 (85.8)	161 (14.2)
**Annual family income**	**≤ U$ 5,472**	1282 (59.4)	1095 (85.4)	187 (14.6)
	**≥ U$ 26,448**	876 (40.6)	752 (85.8)	124 (14.2)
**Semester**	**First**	1260 (56.9)	1092 (86.7)	168 (13.3)
	**Final**	953 (43.1)	811 (85.1)	142 (14.9)
**Type of institution**	**Federal**	554 (25)	463 (83.6)	91 (16.4)
	**State**	808 (36.5)	713 (88.2)	95 (11.8)
	**Private**	851 (38.5)	727 (85.4)	124 (14.6)
**Father’s education level**	**None**	87 (4)	71 (81.6)	16 (18.4)
	**Elementary**	1047 (48.4)	890 (85)	157 (15)
	**Middle School**	695 (32.1)	608 (87.5)	87 (12.5)
	**College**	235 (10.9)	204 (86.8)	31 (13.2)
	**Postgraduate**	100 (4.6)	89 (89)	11 (11)
**Mother’s education level**	**None**	65 (3)	55 (84.6)	10 (15.4)
	**Elementary**	829 (38.3)	704 (84.9)	125 (15.1)
	**Middle School**	787 (36.3)	680 (86.4)	107 (13.6)
	**College**	301 (13.9)	262 (87)	39 (13)
	**Postgraduate**	184 (8.5)	161 (87.5)	23 (12.5)
**Parents’ marital status**	**Married**	1559 (74.2)	1352 (86.7)	207 (13.3)
	**Divorced**	543 (25.8)	459 (84.5)	84 (15.5)

**PTSD:** Posttraumatic Stress Disorder.

The education level of the students’ parents is predominantly at basic school level (elementary and middle school), and is inversely proportional to the probability of their offspring having PTSD. On the other hand, family annual income was in the majority at or below U$ 5,472 (59.4%), and was not clearly associated with PTSD levels.

The majority of students’ parents are married (74.2%), and PTSD is more common among divorced parents’ offspring (15.5% compared to 13.3% of the married parents’ offspring).

Students in federal institutions showed a higher prevalence of PTSD (17%), followed by private institutions (with 14.4% of their students presenting PTSD) and state institutions (with 11.8% presenting), as seen in Table 1.

The median for frequency of trauma exposure was 5 events. The events listed by THQ were grouped into 8 categories: victims of non-sexual violence, sexual violence, accidents, natural disasters, man-made disasters, disease, to witness or receive news of the death or injury of others and other traumas (Table 2). While to witness or receive news of death, acute disease or severe injury of close friends, including family, appeared as the most frequent type of traumatic event (81.7% of the students had experienced this), followed by non-sexual violence (63.9%), and accidents (27.4%), sexual violence was the type of event that most frequently resulted in PTSD (34.1% of the victims developed PTSD).

**Table 2:** Exposure to traumatic events of college students in Northeastern Brazil.

Traumatic Events	Total N (%)	Without PTSD N (%)	With PTSD N (%)
**Victims of non-sexual violence**	1414 (63,9)	1176 (83.2)	238 (16.8)
*Someone tried to take something directly from the subject by using* *force or the threat of force*	785 (35.5)	648 (82.5)	137 (17.5)
*Attempted or actually robbed*	583 (26.3)	477 (81.8)	106 (18.2)
*Attempted or actually had home broken when the subject wasn’t there*	356 (16.1)	294 (82.6)	62 (17.4)
*Attempted or actually had home broken when the subject was there*	255 (11.5)	205 (80.4)	50 (19.6)
*Was attacked with a gun, knife or some other weapon.*	122 (5.5)	89 (73)	33 (27)
*Was someone ever attacked without a weapon and was seriously injured*	55 (2.5)	35 (63.6)	20 (36.4)
*Someone in subject’s family was ever beaten, struck or pushed hard* *enough to cause injury*	291 (13.1)	203 (69.8)	88 (30.2)
**Victims of sexual violence**	208 (9.4)	137 (65.9)	71 (34.1)
*Was ever forced to have intercourse, oral or anal sex against will*	59 (2.7)	35 (59.3)	24 (40.7)
*Someone ever touched the private parts of the body, or made the* *subject touch his/hers, under force or threat*	120 (5.4)	79 (65.8)	41 (34.2)
*Any other situation in which another person tried to force the* *subject to have unwanted sexual contact*	84 (3.8)	53 (63.1)	31 (36.9)
**To witness or receive news of death or injury of others**	1808 (81.7)	1519 (84)	289 (16)
*Witnessed someone seriously injured or killed*	833 (37.6)	681 (81.8)	152 (18.2)
*Exposed to dead bodies*	638 (30.6)	556 (82)	122 (18)
*Close friend or family member was murdered, or killed by a drunk driver*	254 (11.5)	204 (80.3)	50 (19.7)
*Death of a spouse, romantic partner, or child*	35 (1.6)	21 (60)	14 (40)
*Received news of a serious injury, life-threatening illness or unexpected* *death of someone close*	1489 (67.3)	1248 (83.8)	241 (16.2)
**Victims of accidents**	607 (27.4)	492 (81.1)	115 (18.9)
**Victims of natural disaster**	137 (6.2)	97 (70.8)	40 (29.2)
**Victims of man-made disaster**	237 (10.7)	191 (80.6)	46 (19.4)
*Exposed to dangerous chemicals or radioactivity*	86 (3.9)	65 (75.6)	21 (24.4)
*Combat while in military service*	17 (0.8)	17 (100)	0
*Other man-made disaster*	147 (6.6)	121 (82.3)	26 (17.7)
**Victims of disease**	228 (10.3)	169 (74.1)	59 (25.9)
**Victims of other traumas**	232 (10.5)	190 (81.9)	42 (18.1)

**PTSD:** Posttraumatic Stress Disorder.

PTSD prevalence had a strong association with attempted suicide (21.1% of PTSD subjects had already attempted suicide one or more times, compared with 5.4% of non-PTSD subjects). An association between PTSD and high-risk sexual behavior was also found (26.3% of PTSD subjects did not regularly use a condom with non-stable partners). However, substance misuse was not clearly associated with PTSD (Table 3).

**Table 3:** Clinical characteristics of college students with PTSD in Northeastern Brazil.

Clinical Variables		Total N (%)	Without PTSD N (%)	With PTSD N (%)
**PTSD**		2213 (100)	1903 (86)	310 (14)
**Attempted Suicide**		167 (7.6)	102 (5.4)	65 (21.1)
**Alcohol Use**	**Sporadic use**	999 (50)	849 (49.2)	150 (55.4)
	**Weekly use**	282 (14.2)	241 (14.1)	41 (15.1)
	**Daily use**	9 (0.5)	8 (0.5)	1 (0.4)
**Cigarette Use**	**Sporadic use**	175 (8)	143 (7.6)	32 (10.6)
	**Weekly use**	28 (1.3)	22 (1.2)	6 (2)
	**Daily use**	47 (2.2)	35 (1.9)	12 (4)
**Marijuana Use**	**Sporadic use**	93 (4.2)	76 (4)	17 (5.5)
	**Weekly use**	9 (0.4)	9 (0.5)	0 (0)
	**Daily use**	10 (0.5)	7 (0.4)	3 (1)
**Ecstasy Use**	**Sporadic use**	29 (1.3)	25 (1.3)	4 (1.3)
	**Weekly use**	0 (0)	0 (0)	0 (0)
	**Daily use**	1 (0)	1 (0.1)	0 (0)
**Cocaine Use**	**Sporadic use**	41 (1.9)	32 (1.7)	9 (2.9)
	**Weekly use**	5 (0.2)	4 (0.2)	1 (0.3)
	**Daily use**	2 (0.1)	2 (0.1)	0 (0)
**Crack Use**	**Sporadic use**	2 (0.1)	2 (0.1)	0 (0)
	**Weekly use**	0 (0)	0 (0)	0 (0)
	**Daily use**	1 (0)	1 (0.1)	0 (0)
**Condom non-use**	**(non-stable partners)**	363 (16.7)	283 (15.1)	80 (26.1)
**BIS 11**	**Less impulsive**	722 (32.6)	661 (34.7)	61 (19.7)
	**Averagely impulsive**	704 (31.8)	629 (33.1)	75 (24.2)
	**More impulsive**	787 (35.6)	613 (32.2)	174 (56.1)

**PTSD:** Posttraumatic Stress Disorder.

From a total of 3701 students matriculated at the university, 937 (25.4%) were not included due to absence, and 175 (4.8%) were not included because they were younger than 18.

Data regarding 331 (35.3%) of the 937 absent students (matriculated at the university and not encountered in classes), from five of the seven college institutions studied, showed that 56.8% of absent students were matriculated in their first semester, 52.2% were female, 59.2% were 22 years or older, and 84.2% were not local students (Table 4).

**Table 4:** Comparison between present and absent college students.

Socio-Demographic Variables		Present Students N (%)	Absent Students N (%)
**Semester**	**First**	1260 (56.9)	188 (56.8)
	**Final**	953 (43.1)	143 (43.2)
**Gender**	**Female**	1412 (66.1)	109 (52.2)
	**Male**	725 (33.9)	100 (47.8)
**Area**	**Exact Sciences**	278 (12.9)	34 (10.3)
	**Health Sciences**	503 (22.7)	115 (34.7)
	**Human Sciences**	1412 (64.4)	182 (55)
**Origin**	**Local**	844 (42.7)	33 (15.8)
	**Non-local**	1131 (57.3)	176 (84.2)
**Age**	**≤22 years**	1138 (51.4)	135 (40.8)
	**>22 years**	1075 (48.6)	196 (59.2)

Of those 331 absent students, 122 (36.9%) were not present at the time of collection, but there were records of their presence in other classes, 197 of the absent students (59.5%) were matriculated, but there were no records of their presence, and only 12 of the absent students (3.6%) had officially interrupted the semester in progress.

## Discussion

The existing literature has certainly shown progress in understanding the effects of traumatic experiences on mental health, although the issue remains under-researched in low and middle income countries. In Brazil there are just a few studies about PTSD prevalence in selected samples [24], and only two recent, large studies from a general population, both in Southeastern Brazil, the most developed region in Brazil [25], [26]. To our knowledge, this is the first population-based study to investigate trauma exposure and PTSD in Brazilian college students and the first in the Northeastern region of the country. It is plausible that the chosen population is representative of Northeastern college students given that 57.3% were attending university outside of their native cities, which reinforces the idea that migration is an intense phenomenon in this region.

In accordance with previous literature [1], [27], women were more likely to develop PTSD than men (16.2% of woman presented PTSD, compared to 9.9% of men).

The prevalence of PTSD found in this research (14%) is comparable with very high rates found in other studies conducted in low income countries, where people have experienced war, conflict or mass violence (15.8% in Ethiopia, 17.8% in Gaza Strip, 28.4% in Cambodia, and 37.4% in Algeria) [28]. Unsurprisingly, 63.9% of the students reported that they had been exposed to non-sexual violence, and 9.4% of them had been exposed to sexual violence.

It is noteworthy that variation in family annual income was not associated with different PTSD rates, but the educational level of the students’ parents has an inversely proportional association with the students’ PTSD frequency. There is consistent evidence about the role that parents have on the mental health of their offspring [29], and previous studies have already established that a low education level of the victim is a vulnerability factor for PTSD [30]. However, little has been studied about the relationship between parental education level and its effect on the exposure of their offspring to potentially traumatic situations and the subsequent development of PTSD. In the present study, mothers presented a higher level of education (22.4% of them have graduated from college or better), but a lower education level of the father was associated with higher prevalence of PTSD (fathers with no formal education had 18.4% of their offspring presenting PTSD, while fathers with postgraduate degrees had 11% of their offspring with PTSD). One possible hypothesis is that fathers with a low education level may inflict punishment through violence more often and/or with more physical injury than mothers.

A propos, Bordin et al. (2006) [31], executed the first population-based study in Brazil regarding the connection between severe physical punishment and mental health problems in children and adolescents in low income areas, usually associated with low levels of formal education. The research showed that severe punishment is common in this population (10.1%), and that traumatic experiences of infants lead to permanent deficits in the regulation of behavioral, cognitive and emotional processes. This could be a factor that contributes to the intergenerational transmission of violent behaviors, which may in turn be helping to perpetuate the epidemic level of violence.

While to witness or to receive news of death, acute disease or severe injury of close friends, including family, appeared as the most frequent event, its victims presented the lowest rate of PTSD (16%). One hypothesis is that this kind of etiologic event can generate what is known as Partial PTSD [32]. In their investigative study about the concept of Partial PTSD, Breslau et al (2004) [32] demonstrated that while 68.4% of Full PTSD subjects were victims of interpersonal violence, only 46.2% of Partial PTSD subjects were victims of the same type of event, concluding that Partial PTSD is normally due to an etiologic event of lesser magnitude, with different presentation and duration of symptoms.

The present study demonstrated that 21.1% of subjects among the PTSD group have attempted suicide (AS), while the rate in the group without PTSD was almost four-fold lower (5.4%). This is critical information since previous AS is a predictor of additional attempts and of death from a completed suicide [33]–[35]. Therefore, AS has been pointed out as one of the expected consequences of PTSD [36].

BIS-11 demonstrated that increased impulsive behaviors were directly associated with a higher prevalence of PTSD and substance misuse, which is well established by previous studies. [37], [38]. However, we did not observe an association between PTSD and substance misuse. This finding is not in accordance with previous reports in the literature [1]. More studies are necessary to better explore this finding and to better understand which other factors could be interfering, such as sub notification of cases or report bias.

High-risk sexual behavior was more frequent among PTSD subjects, as well as among the more impulsive participants: 49.5% of the most impulsive subjects of the study did not regularly use a condom with non-stable sexual partners (compared to 18.9% of the less impulsive ones, when BIS-11 is divided in tertiles). Both outcomes are in accordance with previous literature [39], [40].

Data regarding the absent students (those matriculated at the university and not encountered in class) showed that both present and absent students have a similar profile based on current semester, gender and area of study. An important difference between present and absent students is shown by the variable *origin*, and there is some difference with regard to the *age* variable. Neither of these two variables in the enrolled group was associated with a clinically meaningful elevation of PTSD rates.

No records were found regarding the reasons for interrupting the semester or for absenteeism. One possible reason for the age variation between groups is increased absence due to a conflicting employment, especially for those older students who no longer have any parental support. Another possible reason is the missing of classes due to difficulties with public transportation (especially those who live in surrounding areas), as well as frequent trips, especially by non-local students whose parents live outside the metropolitan area. Matriculation at multiple college institutions, for first semester students is another possible explanation. This is a common phenomenon in Brazilian universities, where students compete in several exams (potentially in multiple states), with results being released at different times, and matriculate in each one to keep their options open.

### Limitations

The non-inclusion of absentee and non-consenting students in the analyses may have resulted in the loss of the most impulsive and/or most severely affected individuals. Also, other outcomes of trauma exposure that can act as confounders were not investigated, such as depressive or other symptoms of anxiety and thus were not controlled for. On the other hand, avoidance is a well-known phenomenon in PTSD, indeed being one of its diagnosis criteria [41], and may result in less accurate reports. It is assumed that self-applied scales can reduce the report bias. In addition, memory bias can also occur, resulting in less reliable reports, given that some events may have occurred in the early life of respondents.

## Conclusions

To the best of our knowledge, this is the first study to investigate trauma exposure and PTSD prevalence in Brazilian college students, a non-clinical population. It shows a high frequency of exposure to violence, as well as a high rate of PTSD conversion, suicide attempts, and high-risk sexual behavior. This highlights the importance of effective public health actions in relation to primary and secondary prevention and treatment of PTSD and other dysfunctional behaviors resulting from traumatic exposure among Brazilian youth, usually an at-risk population for violence and traumatic situations.

It is also very important that the primary service professionals receive adequate training to screen and identify traumatic life events. Since traumatized patients tend to manifest avoidance behavior, they may underreport traumatic experiences if not interrogated. The delay in proper diagnosis of PTSD may contribute to increased distress and symptoms, which can be a risk factor for the development of comorbidities or death.

## Acknowledgments

Adriana Duarte, Amanda Loureiro, Deivson Mundim, Rejane Santana, Isabela Albuquerque, Rana Barbosa, Lilian Carvalho, Susan Carvalho, Reinilza Nunes, Weslley Batista, Maria Rosa Dantas, Antonio Dantas Júnior, Albaniza Formiga, Fernanda Dias, Fransuélio Nascimento, Jesana Damasceno, Perla Soares, and Rubens Lima.

## Supporting Information

Figure S1 **Flowchart of data collection.**
(TIFF)
