# Supplementary figures and images for: Clinical and Socio-Demographic Characteristics of College Students Exposed to Traumatic Experiences: A Census of Seven College Institutions in Northeastern Brazil
Source: PLoS One. 2013 Nov 13;8(11):e78677. doi: 10.1371/journal.pone.0078677 (PMC3827274; doi:10.1371/journal.pone.0078677)

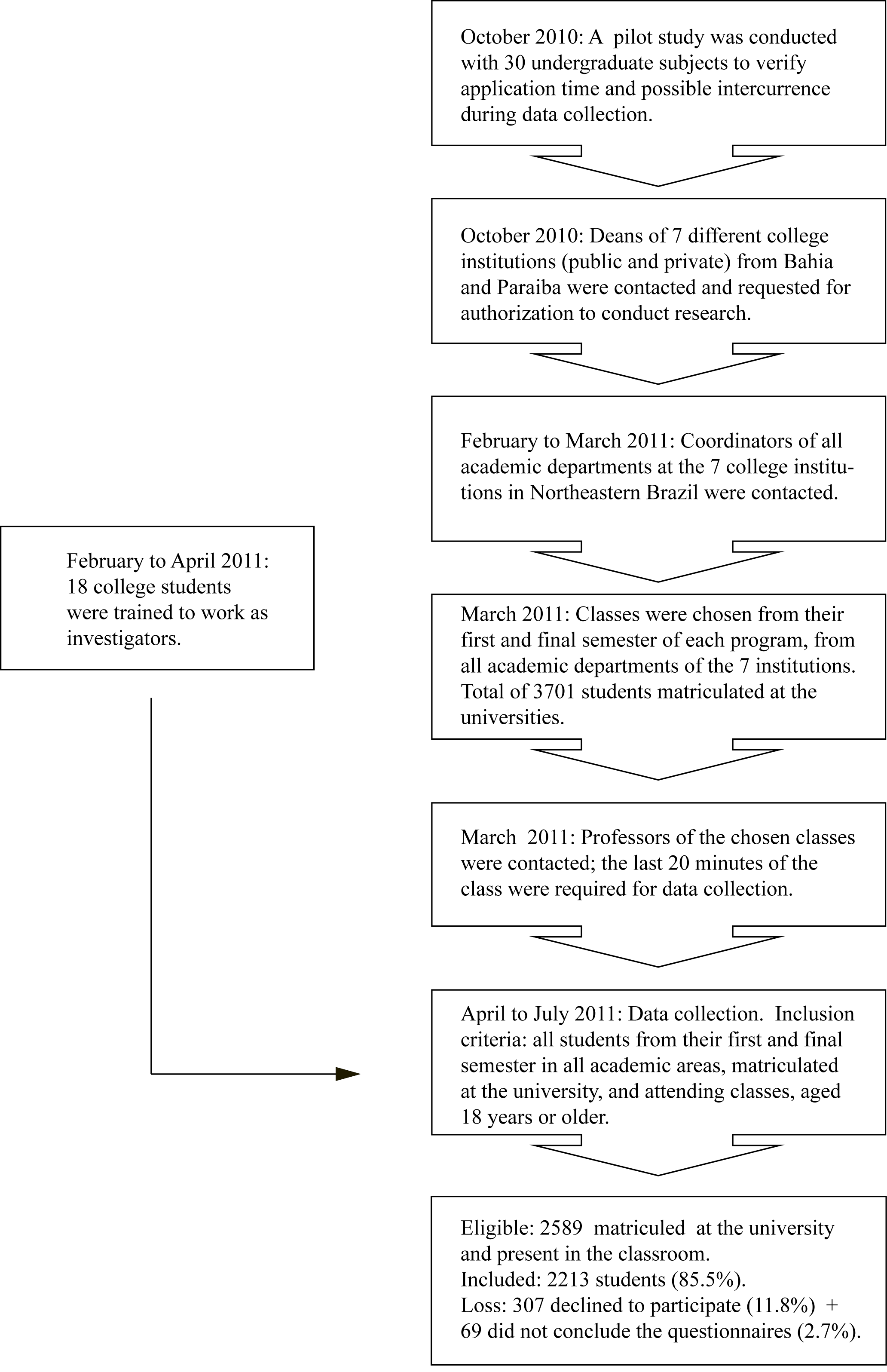

Supplement: Figure S1 — Flowchart of data collection. (TIFF) [file pone.0078677.s001.tiff]
